# Quantification of Trace Mercury in Water: Solving the Problem of Adsorption, Sample Preservation, and Cross‐Contamination

**DOI:** 10.1002/gch2.201900061

**Published:** 2019-11-11

**Authors:** Jingqi Zhang, Jingbo Chao, Yang Tang, Pingyu Wan, Xiao Jin Yang, Choon Wong, Mark Bruce, Qing Hu

**Affiliations:** ^1^ Beijing Key Laboratory of Membrane Science and Technology and Department of Environmental Science and Engineering Beijing University of Chemical Technology Beijing 100029 China; ^2^ National Institute of Metrology Beijing 100029 China; ^3^ School of Chemistry Beijing University of Chemical Technology Beijing 100029 China; ^4^ NSW Forensic & Analytical Science Service 480 Weeroona Rd Lidcombe NSW 2141 Australia; ^5^ Eurofins TestAmerica 4101 Shuffel St. NW North Canton OH 44720 USA; ^6^ Southern University of Science and Technology Shenzhen Guangdong 518055 China

**Keywords:** adsorption, cross‐contamination, mercury solution, stability, water samples

## Abstract

Adsorption, sample preservation, and cross‐contamination are the major impediments to the accurate and sensitive analysis of low‐level mercury samples. Common measures to deal with this issue are to use Teflon, quartz, or borosilicate glass bottles for sampling, standard solution and sample preservation with oxidative chemicals, to prepare standard solutions daily and to use dedicated glassware. This paper demonstrates that these measures are neither efficient nor effective. Two common laboratory sample containers (borosilicate volumetric glass flasks and polypropylene tubes) are investigated for the preparation and preservation of water samples and standard solutions of 0.2–1 µg L^−1^ with 2% HNO_3_. Mercury adsorption rates of 6–22% are observed within 30 min and after 48 days, the adsorption is greater than 98%. In stark contrast, no adsorption is found during a testing period of 560 days when the solutions are subject to potassium permanganate‐persulfate digestion. New glass flasks and polypropylene bottles are free of mercury contamination but reused flasks are a major source of mercury cross‐contamination. To minimize adsorption and cross‐contamination, standard solutions are treated by potassium permanganate‐persulfate or BrCl digestion, and each individual sample and standard solution should be stored and prepared in single‐use polypropylene bottle, without transference.

## Graphical abstract

Adsorption and cross‐contamination are the major challenges for accurate analysis of trace mercury. This study demonstrates that adsorption is the root cause for cross‐contamination, and single use of polypropylene plastic bottles for sample preparation and storage is highly efficient to completely solve these notorious problems as long as sample and standard solutions are subject to potassium permanganate‐persulfate digestion.

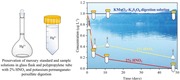

## 1 Introduction

Mercury pollution is one of the world's major environmental threats and its toxicity and damage to human health, in particular to young children, has been clearly established.^1^ Global mercury emissions have surged since the 1950s and currently human activities result in mercury emissions of 2000 metric tons per year.^2^ Bioaccumulation of mercury in fish is well recognized and hazards associated with eating fish have become public health concerns.^3^ A typical example of the impact of mercury pollution on humans is Minamata disease discovered in Japan in 1956 (2265 victims were officially recognized and 1784 of whom died). High concentrations of mercury in North American freshwater fish have prompted health authorities in Canada and most U.S. States to warn against eating too much fish.^4^ A full understanding of mercury toxicity and damage to the environment and living organisms is dependent on the accurate and sensitive analysis of mercury and its speciation.^5^

The common laboratory practice for the preservation of sample and calibration standard solutions for trace metal analysis is to use volumetric glass flasks and/or high‐density polyethylene (HDPE) plastic bottles and 2% v/v HNO_3_. For most elements, standards and samples at µg L^−1^ and mg L^−1^ with 2% v/v HNO_3_ are stable for months. However, mercury standards and samples are an exception. Significant losses of mercury from µg L^−1^ solutions have been observed regardless of container materials (typical examples are summarized in **Table** **1**).^6^ The proposed mechanisms for this instability of mercury standard and sample solutions have included adsorption on the container's interior wall, mercury volatilization/permeation and conversion of mercury species.[qv: 6d,7] Parker and Bloom reported that acidification of environmental waters with 2% HNO_3_ may cause coagulation of natural organic matter (NOM) on to which mercury may be adsorbed.[qv: 6d] The adsorbed Hg^2+^ can be reduced to Hg^+^ and/or Hg^0^ (elemental mercury) in the presence of a reducing agent occurring either naturally (i.e., micro‐organisms, humic acids^8^) or as impurities in solution^9^ or by photoreduction.^10^ Hg^+^ could then disproportionate spontaneously producing Hg^2+^ and elemental mercury.^9^, ^11^ Consequently, elemental mercury is lost by permeation and diffusion through the wall of plastic bottles[qv: 6d,e,j,9,12] and volatilization and diffusion through the threads of the bottle cap.^13^

**Table 1:** Instability of mercury samples and solutions reported in the literature (1972–2013)

Sample type	Container ^a)^	Hg Conc. [µg L^−1^]	Preservation	Mercury loss rate	Year [Ref.]
Distilled water	PE	50	pH 1, HCl	100% in 10 days	1972[qv: 6c]
Creek water	PE	50	pH 1, HCl	80% in 10 days	1972[qv: 6c]
Distilled water	PE	25	2% HNO_3_	75% in 150 h	1973[qv: 6g]
Distilled water	Glass	1	1% HNO_3_	70% in 10 days	1974[qv: 6e]
Distilled water	PE	1	5% HNO_3_	75% in 15 days	1979[qv: 6f]
Distilled water	PE	1	1% HNO_3_	75% in 15 days	1983[qv: 6b]
Distilled water	PE	30	0.5 m HNO_3_	100% in 50 days	1988[qv: 6i]
Distilled water	PE	4	1% HNO_3_	87% in 12 days	1990[qv: 6j]
Potable water	Glass	1	2% HCl	40% in 10 days	1996[qv: 6h]
Lake water	Teflon	0.01–0.4	2% HNO_3_	50% in 10 days	2005[qv: 6a]
Distilled water	PP	1	10% HNO_3_	94% in 7 days	2005[qv: 6d]
River Water	Teflon	0.003–0.013	1% HCl	70% in 7 days	2013^14^

^a)^ PE, PP, and HDPE denote polyethylene, polypropylene, and high‐density polyethylene.

The mechanism for the loss of mercury from solution has long been a subject of debate.[qv: 6d,7] For instance, the adsorption mechanism is unable to explain why similar rates of adsorption have been observed for containers of different materials (Table 1). Feldman[qv: 6e] observed 70% losses of mercury from 1 µg L^−1^ in 1% HNO_3_ in 250 mL glass flasks in 10 days while Heiden and Aikens[qv: 6b] reported 75% losses for similar solutions in 500 mL PE containers in 15 days. We also observed similar rates of mercury losses from µg L^−1^ solutions preserved in 50 mL volumetric glass flasks and PP centrifuge tubes. Creswell et al. observed positive biases of total mercury analysis from the original collection bottle after a sub‐sample was removed for other analyses and identified that the positive biases were attributed to the adsorption of mercury on the interior wall of the original collection bottle.[qv: 7b] It is generally recognized that Teflon, quartz, and borosilicate glass have lower tendencies to adsorb mercury than polyethylene (PE) and polypropylene (PP) plastics,[qv: 6i,j,14] although considerable losses of mercury in quartz, Teflon, and glass containers have also been observed[qv: 7a] (Table 1).

To minimize the adsorption losses, the APHA and U.S. EPA standard methods require that standard calibration mercury solutions at µg L^−1^ and lower be prepared daily with 2% HNO_3_ and that water samples be preserved with HNO_3_, HCl, BrCl, KBrO_3_, or KBr in HDPE, glass or fluoropolymer bottles on collection or within 48 h of collection.^15^ Nevertheless, substantial losses of mercury in quartz and glass containers were still observed within 30 min from preparation.[qv: 7a] In addition, handling and manipulation of strong acids or oxidative, hazardous chemicals in field sampling pose safety and environmental risks.[qv: 15e,f]

To reduce the risks of mercury contamination, the U.S. EPA standard methods require that at least 5% of the bottles from a given lot should be tested by filling with reagent water acidified to pH < 2, standing for a minimum of 24 h and then analyzing for contamination (24 h acid filling test).[qv: 15e,f] However, we often observed mercury contamination for glass flasks after sample digestion even though the flasks had been identified as not contamination by the 24 h acid filling test prior to sample digestion. Parker and Bloom found that new glass flasks were free of mercury contamination.[qv: 6d] However, the use of new volumetric glass flasks for each sample and standard solution is a very costly imposition for routine analysis and testing.

**Figure 1:**
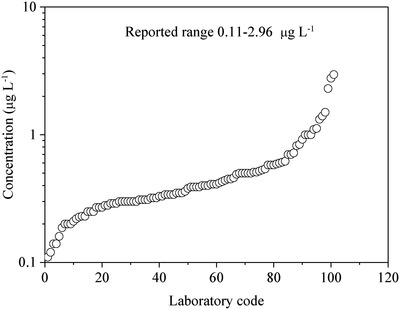
Reported mercury concentrations of a water sample preserved in 2% HNO_3_ in borosilicate glass bottle in a proficiency study organized by the National Authority of Testing Association (NATA). The number of participating laboratories was 122, of which six labs reported < 0.1 µg L^−1^, two labs < 0.3 µg L^−1^, eight labs < 0.5 µg L^−1^, two labs < 1 µg L^−1^, and two labs < 2 µg L^−1^ and one lab < 6 µg L^−1^. The reported concentration ranged between 0.11 and 2.96 µg L^−1^.

The accurate determination of mercury concentrations at low levels has long been a challenge[qv: 5d,e,7a,14,16] and the problem is illustrated by an inter‐laboratory proficiency study of trace mercury analysis in water (**Figure** **1**), which demonstrates the wide variability of determinations. It is generally recognized that the instability of mercury solutions and mercury cross‐contamination are the two major causes for the large errors in trace mercury quantification. This study is aimed at investigating the stability of mercury solutions and samples preserved in glass flasks and PP centrifuge tubes and the elimination of mercury cross‐contamination to improve the accuracy of trace mercury quantification.

## 2 Results and Discussion

**Figure 2:**
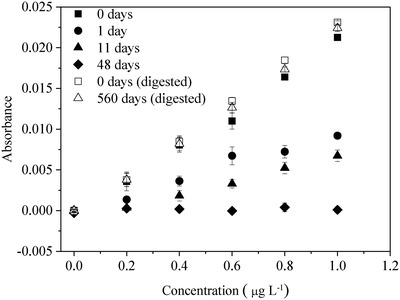
Effect of solution storage time on the Hg determination of 0.2–1 µg L^−1^ mercury solutions in PP tubes. Solutions were prepared with 2% HNO_3_ by dilution from a commercial stock solution (20 mg L^−1^ Hg in 4% HNO_3_) and directly analyzed by CVAAS or treated by the KMnO_4_–K_2_S_2_O_8_ digestion and then analyzed by CVAAS.

As discussed previously, considerable losses of mercury from µg L^−1^ solutions (2% HNO_3_) were observed within 30 min from preparation.[qv: 7a] Indeed, we observed 6–22% losses of mercury from standard solutions of 0.2–1 µg L^−1^ in 2% HNO_3_ from 50 mL volumetric glass flasks within 30 min from preparation. The solutions lost 36–43% of mercury from 50 mL PP centrifuge tubes in one day and the losses increased with standing time of the solution, reaching 98% by 48 days (**Figure** **2**). Overall, no significant differences were observed in the stability of standard mercury solutions (0.2–1 µg L^−1^ in 2% HNO_3_) stored in glass flasks and PP tubes while the adsorption rates observed here were similar to the results reported in the literature (Table 1). Interestingly, mercury solutions were quite stable in the 50 mL PP tubes during the test period of 560 days following KMnO_4_–K_2_S_2_O_8_ digestion (Figure 2). The treated solutions were tightly capped and placed on the laboratory bench. This observation indicates that the container material (glass or PP), light radiation and seasonal variations in ambient room temperature had no influence on the stability of mercury solutions after the KMnO_4_–K_2_S_2_O_8_ digestion.

**Figure 3:**
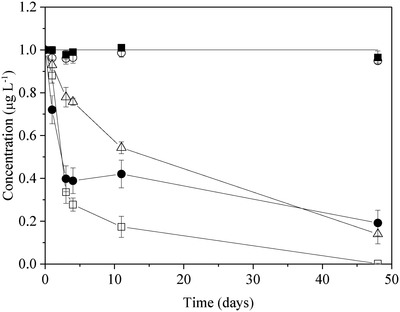
Concentrations of 1 µg L^−1^ mercury solutions as a function of storage time. Solutions were prepared with 2% HNO_3_ or the KMnO_4_–K_2_S_2_O_8_ digestion solution and stored in “un‐deactivated (new)” and “deactivated” containers. ◾ = new PP, digestion solution, ○ = “deactivated” PP, digestion solution, △ = “deactivated” PP, 2% HNO_3_, ⚫ = “deactivated” glass, 2% HNO_3_, □ = new PP, 2% HNO_3_.

The KMnO_4_–K_2_S_2_O_8_ digestion is designed to oxidize all Hg species to inorganic Hg^2+^ and therefore should have no effect on the Hg^2+^ in the standard mercury solutions, which are prepared by simply diluting the stock solution (20 mg L^−1^ in 4% HNO_3_) with 2% HNO_3_. In this context, the long‐term stability of mercury solutions after the KMnO_4_–K_2_S_2_O_8_ digestion was because the digestion deactivates the interior surface of containers, thus preventing adsorption of mercury. To verify this hypothesis, the glass flasks and PP tubes were treated with the KMnO_4_–K_2_S_2_O_8_ digestion to “deactivate” the interior surface of the flasks and tubes and then mercury solutions were added for the stability test. The solutions prepared with 2% HNO_3_ in the “deactivated” glass flasks and PP tubes lost greater than 65% of mercury after 48 days whilst the solutions prepared with the “digestion solution” were stable both in “un‐deactivated” and “deactivated” containers (**Figure** **3**). Thus the KMnO_4_–K_2_S_2_O_8_ digestion can be seen to play no role in deactivating container surfaces.

The oxidative chemicals (K_2_Cr_2_O_7_, KBrO_3_, KMnO_4_, or BrCl) are designed to maintain mercury in the form of Hg^2+^ in an oxidizing environment.[qv: 6e,i,7a,12d,15e,f] Clearly, mercury in the solutions prepared with “digestion solution” was not in an oxidizing environment because the oxidative chemicals KMnO_4_ and K_2_S_2_O_8_ are completely reduced by the presence of the excess reducing agent, hydroxylamine hydrochloride (see Section 4, Experimental Section). The digestion solutions consisted of K^+^, SO_4_^2−^, Mn^2+^, Cl^−^, NH_4_^+^, and HONH_3_Cl and are of high ionic strength and it is possible that the presence of these ions prevents the adsorption of mercury. Indeed, inorganic ions such as Ca^2+^, Mg^2+^, SO_4_^2−^, HCO_3_^−^, Na^+^, and K^+^ could occupy the active sites of the container's interior surface, thus reducing the rate of mercury adsorption.[qv: 7a]

**Figure 4:**
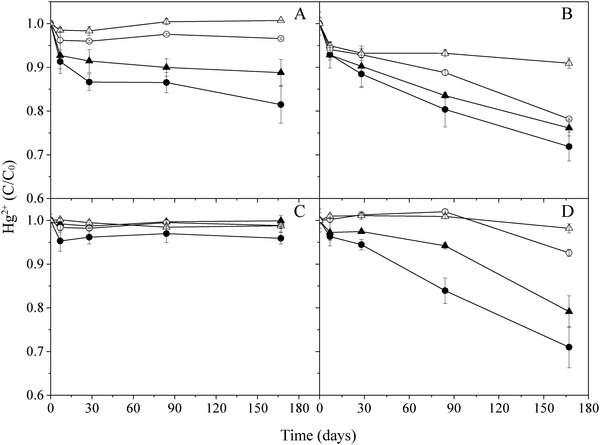
Stability of 1 µg L^−1^ mercury solutions preserved with different concentrations of NaCl and NaNO_3_ in 50 mL volumetric glass and PP (polypropylene) flasks. A) NaNO_3_‐Glass, B) NaNO_3_‐PP, C) NaCl‐Glass, D) NaCl‐PP. Salt concentration: ⚫ = 0.1%, ▲ = 0.3%, ○ = 1%, △ = 3%.

To examine the effect of ionic strength on mercury stability, we added NaCl and NaNO_3_ to the solutions (**Figure** **4**). The stability of mercury increased with increasing ionic strength (i.e., salt concentration). Mercury was more stable in NaCl solutions than in NaNO_3_ solutions and mercury solutions prepared with NaCl and NaNO_3_ were more stable in glass containers than in PP plastic containers. The increased stability of mercury with increased NaCl concentration has been observed previously.^17^ Considering the stabilizing effect of NaCl and NaNO_3_, we also investigated the desorption of mercury from the interior surface of 50 mL glass volumetric flasks and 50 mL PP tubes by NaCl and NaNO_3_. The samples of 25 mL 2% HNO_3_ solution containing 0.05 µg Hg (i.e., 2 µg L^−1^) in 50 mL glass flasks and PP tubes were placed on laboratory bench. After 28 days, 25 mL 6% NaCl or 6% NaNO_3_ was added and then equilibrated for 7 days prior to CVAFS measurement. We found that 94 ± 2% and 74 ± 3% of mercury was recovered from glass by NaCl and NaNO_3_, respectively and much lower recoveries (54 ± 1% and 53 ± 1%) from the PP tubes. Therefore, the addition of salts (i.e., increasing ionic strength) is able to desorb mercury from glass and plastic PP containers. In this context, it would be interesting to see whether the KMnO_4_–K_2_S_2_O_8_ digestion is able to quantitatively recover mercury from the interior surface of sample containers, in particular from PP containers.

**Figure 5:**
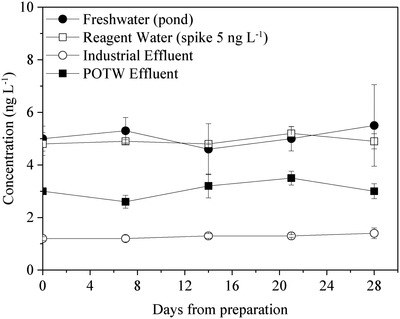
Mercury stability in environmental water samples. The water sample was unpreserved at time of sampling and the digestion with BrCl was carried out in the original sampling bottle (borosilicate glass). Three samples were prepared for each type of water. Method 1631C[qv: 15d] was used for preparation and analysis.

**Table** **2** shows mercury recovery of 88–110% from de‐ionized, tap and creek water samples. These samples were stored in glass flasks and PP tubes without preservatives, placed on the laboratory bench for 21 days and then digested in the original flasks and tubes by the KMnO_4_–K_2_S_2_O_8_. The results indicate that the KMnO_4_–K_2_S_2_O_8_ digestion quantitatively recovered mercury adsorbed on the interior surface of both glass flasks and PP tubes, because at least 60% of the mercury would have been adsorbed from such unpreserved samples in accordance with previous results (Figure 2). We also observed that mercury was quantitatively recovered in unpreserved environmental water samples in glass bottles within 28 days from sampling when the BrCl digestion was performed in the original sampling glass bottles (**Figure** **5**). The above results demonstrate that sample preservation with acid or oxidative chemicals is not necessary in field sampling if the KMnO_4_–K_2_S_2_O_8_ or BrCl digestion is performed in the original sampling bottles (glass and PP) prior to total mercury analysis.

**Table 2:** Quantification of trace mercury in water samples preserved with 2% HNO_3_ in volumetric borosilicate glass flasks and PP tubes for 21 days by CVAAS

Sample type ^a)^	Mercury concentration [µg L^−1^]^b)^	
	Borosilicate glass flasks	Polypropylene (PP) tubes
De‐ionized water	0.994 ± 0.081 (99.4%)	0.997 ± 0.095 (99.7%)
Tap water	1.084 ± 0.128 (108%)	0.887 ± 0.022 (88.7%)
Creek water	1.102 ± 0.110 (110%)	0.882 ± 0.015 (88.2%)

^a)^ The samples (25 mL containing 0.05 µg Hg) were prepared with de‐ionized, tap and creek water, preserved with 2% HNO_3_ in 50 mL volumetric borosilicate glass flasks and 50 mL PP tubes and placed on laboratory bench. After 21 days, the samples were digested by KMnO_4_–K_2_S_2_O_8_ digestion in the same flasks and tubes and then made up to the mark (50 mL)

^b)^ The results are the average of three separate preparations with standard deviation and percentage recovery.

The above results confirm that adsorption of mercury ions on the interior wall of container is the dominant cause for the loss of mercury from standard solutions and samples. Yu and Yan provided a well‐summarized report on the factors affecting the stability of mercury solutions and samples, including concentration of mercury species, sample composition, container material, storage temperature, pH of sample solution and light.[qv: 7a] In general, the relative adsorption rate decreases with increasing concentration of mercury in the solution.[qv: 7a] Toribara et al. observed that the more dilute Hg(II) solutions would be less stable and lose mercury more readily.^9^ The stock mercury solution employed in this study was 20 mg L^−1^ preserved in HDPE bottle with 4% HNO_3_. The supplier provided a one‐year guarantee of the stability from the date of receipt. Different batches of this mercury stock solution were tightly capped and stored in laboratory cupboards without any other particular protection. We tested the stability of these stock solutions by instant CVAAS measurement after appropriate dilutions and found that the concentration was within an error of 5% over a period of 5 years from date of receipt. Clearly, the long‐term stability of these mercury stock solutions in the HDPE bottles did not support the loss mechanism of mercury via diffusion through the wall of sample containers[qv: 6d,j,9,12] and the threads of sample bottle's cap.^13^ The long‐term stability of these 20 mg L^−1^ solutions was most likely that the amount of mercury adsorbed was insignificant relative to the total amount of mercury from such high concentrations.

Mercury contamination has long been a critical issue in trace mercury analyses[qv: 6d,j,15e,f] and chemicals used for preservation and digestion are the major sources of mercury contamination.[qv: 6j,7a,15b,e,f] In particular, used glass bottles and flasks incur high risks of mercury contamination.[qv: 15e,f] The U.S. EPA recommends contamination checks by filling the bottles with dilute acid (pH < 2), standing for at least 24 h and analyzing the solution,[qv: 15c‐f] while the APHA standard method recommends use of dedicated glassware whenever possible.[qv: 15a] **Table** **3** shows the results for blank testing of used and new volumetric glass flasks. The dedicated flasks used for mercury solutions/samples preparation and storage were cleaned by a typical acid cleaning procedure (see Section 4). Prior to use, these flasks were filled with 2% HNO_3_, placed on a bench for 72 h and then analyzed directly by CVAAS. No mercury contamination was identified (Entry 2, Table 3). However, significantly elevated mercury concentrations were detected in these dedicated flasks following the KMnO_4_–K_2_S_2_O_8_ digestion (Entry 3, Table 3). Initially, we considered that it was possibly due to contamination of the chemicals used and/or digestion procedure. By contrast, we found that the blank values for new glass flasks by the same digestion procedure were negligible (Entry 4, Table 3). Therefore, the high blank values from the used glass flasks following KMnO_4_–K_2_S_2_O_8_ digestion must have been derived from the trace amounts of mercury adsorbed on the interior surface of the flasks. The mercury‐free status of new glassware and the high risk of contamination from used glassware has been previously reported.[qv: 6d]

**Table 3:** Blank values of mercury from used and new 50 mL volumetric glass flasks by CVAAS

Flask No.^a)^	Used glass flasks [µg L^−1^]^b)^		New glass flasks [µg L^−1^]^c)^
	2% HNO_3_ filling test	KMnO_4_–K_2_S_2_O_8_ digestion	KMnO_4_–K_2_S_2_O_8_ digestion
1	0.002	0.047	0.008
2	0.004	0.136	0.001
3	0.003	0.119	0.004
4	0.001	0.130	0.009
5	0.000	0.328	0.004
6	0.001	0.261	0.004
7	0.001	0.058	0.003
8	0.003	0.048	0.003

^a)^ The limit of detection was 0.05 µg L^−1^

^b)^ Eight used and cleaned glass flasks were filled with 2% HNO_3_, placed on laboratory bench for 72 h and analyzed directly by CVAAS. Then, these flasks were rinsed with high‐purity water thoroughly and 25 mL high‐purity water was added to each flask, digested by KMnO_4_–K_2_S_2_O_8_, made up to the mark with high‐purity water (see Section 4) and then analyzed by CVAAS. The *p* value from Wilcoxon signed‐rank tests at 95% level confidence between these two batches of samples is 0.0116 (<0.05), indicating statistically significant differences

^c)^ Eight new glass flasks were rinsed with high‐purity water thoroughly and 25 mL high‐purity water was added to each flask, digested by KMnO_4_–K_2_S_2_O_8_, made up to the mark with high‐purity water and then analyzed by CVAAS. The *p* value between these new flask samples and the used flask‐2% HNO_3_ samples is 0.7789 (>0.05), indicating no significant differences. This test demonstrates that the high blank values for the used flasks‐KMnO_4_–K_2_S_2_O_8_ digestion samples were not from the chemicals used.

## 3 Conclusion and Recommendation

Findings of this work are:

1) The typical cleaning procedure using 40% HNO_3_ soaking overnight is not able to completely remove trace amount of mercury from the interior surface of glass flasks;
2) Glass flasks and bottles for sample preparation and storage incur high risks of mercury cross‐contamination;
3) There are no significant differences in the rates of mercury adsorption from µg L^−1^ solutions in 2% HNO_3_ between volumetric glass flasks and polypropylene tubes;
4) The KMnO_4_–K_2_S_2_O_8_ and BrCl digestion quantitatively desorbs mercury from the interior surface of bottles back into solution;
5) Mercury samples and standard solutions are extremely stable both in glass flasks and PP tubes after the KMnO_4_–K_2_S_2_O_8_ or BrCl digestion;
6) There are no differences in the stability of mercury standards treated by the KMnO_4_–K_2_S_2_O_8_ digestion and prepared by a simple dilution using the KMnO_4_–K_2_S_2_O_8_ digestion blank solutions.

The following recommendations are made to eliminate mercury adsorption and cross‐contamination:

1) Use new PP bottles for field sampling and storage for trace total mercury analysis and do not add additional “preservative” chemicals;
2) Digest, make up and preserve/store the sample in the original sampling bottles;
3) Use new PP bottles for standard solutions and prepare the standards as per samples;
4) Calibrate the instrument using as‐prepared standard solutions until finished (prepare new after 3 months).

## 4 Experimental Section

*Chemicals and Apparatus*: High‐purity water used throughout the study was prepared from Millipore Milli‐Q deionized‐water system (Billerica, MA, USA). Ultrapure concentrated nitric acid (HNO_3_, 69% w/w), analytical grade sulfuric acid (H_2_SO_4_), hydrochloric acid (HCl), stannous chloride (SnCl_2_), hydroxylamine hydrochloride (HONH_3_Cl), potassium permanganate (KMnO_4_), and potassium persulphate (K_2_S_2_O_8_) were obtained from BDH Chemicals, Poole, England. Stock mercury solutions of 20 mg L^−1^ (4% HNO_3_, Alpha Resources, U.S.A.) and 100 µg mL^−1^ (4% HNO_3_, National Institute of Metrology, China) were used for preparation of sample solutions of 0.1–1 µg L^−1^ by dilution with 2% HNO_3_. Cold vapor atomic absorption spectrometer (CVAAS), Perkin Elmer FIMS‐400 with an AS‐90 autosampler or cold vapor atomic fluorescence spectrometer (CVAFS), Beijing Titan Instruments AFS‐9130, China, were employed to measure mercury concentrations (see Section 4, *Analysis with CVAAS or with CVAFS*).

*KMnO_4_–K_2_S_2_O_8_ Digestion*: Mix the sample thoroughly to achieve homogeneity and transfer 25 mL aliquot of the sample into a 50 mL volumetric borosilicate glass flask or 50 mL polypropylene centrifuge tube, add 1 mL of concentrated HNO_3_ and 10 mL of 0.25 mol L^−1^ H_2_SO_4_. Mix the solution after each addition, then add 7.5 mL of 5% w/v KMnO_4_ solution and 2.5 mL of 5% w/v K_2_S_2_O_8_ solution to the flask and mix thoroughly. Place the flask in the water bath for 1 h at 95 °C. Cool and add enough 10% hydroxylamine hydrochloride (HONH_3_Cl) solution (10% w/v) to reduce excess amount of KMnO_4_ and K_2_S_2_O_8_ in solution until the purple color disappears. Dilute to the mark with water and filter through a 0.22 µm membrane (Whatman Filter No. 541), if necessary. When deionized water was used instead of mercury sample, the resultant solution was termed “Digestion solution.”

*BrCl Digestion*: Transfer 100 mL sample into a 125 mL fluoropolymer bottle. For clear water and filtered samples, add 0.5 mL of BrCl; for brown water and turbid samples, add 1.0 mL; for sewage effluent, add 5 mL. Cap the bottle and digest at room temperature for a 12 h minimum (or place sealed bottles in oven at 50 °C for 6 h). Add 0.2–0.25 mL of HONH_3_Cl solution to the BrCl‐oxidized sample, cap the bottle and swirl the sample until the yellow color disappears. Allow the sample to react for 5 min with periodic swirling. Connect a fresh trap to the bubbler, pour the reduced sample into the bubbler, add 0.5 mL of SnCl_2_ solution, and purge the sample onto a gold trap with N_2_ at 350 ± 50 mL min^−1^ for 20 min, prior to CVAFS detection.

*Analysis with CVAAS*[qv: *15a,b*] *or with CVAFS*[qv: *15e,f*]: Mercury was determined by cold vapor atomic absorption spectrometer using tin(II) chloride as reductant at a wavelength of 253.7 nm or cold vapor atomic fluorescence spectrometer using sodium borohydride as reductant. Loss and recovery of mercury from solutions were calculated by

(1) $$\mathrm{Loss}\;\mathrm{rate}\;=\frac{C_{0}-C}{C_{0}}\;\times \;100\%$$

(2) $$\mathrm{Recovery}\;\mathrm{rate}\;=\frac{C}{C_{0}}\;\times \;100\%$$

where *C*_0_ is initial concentration of mercury and *C* is the concentration of mercury after sample processing.

*Preservation and Storage of Mercury Standard Solutions and Samples*: Mercury solutions of 1 µg L^−1^ were prepared in 50 mL volumetric glass flasks or 50 mL PP centrifuge tubes by dilution from stock mercury solutions with 2% HNO_3_ or the digestion blank solution (see Section 4, *KMnO_4_–K_2_S_2_O_8_ Digestion*). For comparing stability of mercury solutions, the glass flasks and PP tubes were “de‐activated” by the KMnO_4_–K_2_S_2_O_8_ digestion and the de‐activated flasks and tubes were used for storing mercury solutions. After preparation, the solutions and samples in glass flasks and PP tubes were capped and placed on the open laboratory bench (i.e., full exposure to ambient light and temperature).

*Cleaning Procedure for Glass Flasks and Bottles*: Glass bottles/flasks were soaked in hot (65–75 °C) concentrated HNO_3_ for at least 48 h. After cooling, they were rinsed three times with reagent water followed by detergent and deionized water wash. Then, they were rinsed three times with reagent water, and placed in a mercury‐free Class‐100 clean bench until the outside surfaces were dry. The cleaned bottles/flasks were tightly capped and stored in plastic boxes until use.

## Conflict of Interest

The authors declare no conflict of interest.
